# Adaptive Evolution of Genes Involved in the Regulation of Germline Stem Cells in *Drosophila melanogaster* and *D. simulans*

**DOI:** 10.1534/g3.114.015875

**Published:** 2015-02-09

**Authors:** Heather A. Flores, Vanessa L. Bauer DuMont, Aalya Fatoo, Diana Hubbard, Mohammed Hijji, Daniel A. Barbash, Charles F. Aquadro

**Affiliations:** Department of Molecular Biology and Genetics, Cornell University, Ithaca, New York 14853

**Keywords:** germline stem cells, adaptive evolution, positive selection, oogenesis, spermatogenesis

## Abstract

Population genetic and comparative analyses in diverse taxa have shown that numerous genes involved in reproduction are adaptively evolving. Two genes involved in germline stem cell regulation, *bag of marbles* (*bam*) and *benign gonial cell neoplasm* (*bgcn*), have been shown previously to experience recurrent, adaptive evolution in both *Drosophila melanogaster* and *D. simulans*. Here we report a population genetic survey on eight additional genes involved in germline stem cell regulation in *D. melanogaster* and *D. simulans* that reveals all eight of these genes reject a neutral model of evolution in at least one test and one species after correction for multiple testing using a false-discovery rate of 0.05. These genes play diverse roles in the regulation of germline stem cells, suggesting that positive selection in response to several evolutionary pressures may be acting to drive the adaptive evolution of these genes.

Reproduction and fertility are among the most important traits for organismal fitness. Many models and theoretical studies have proposed that germline and fertility-related genes will be targeted for selection, and empirical evidence has documented rapid evolution and in many cases positive selection on numerous genes known or proposed to be involved in male fertility (Tsaur *et al.* 1998; Begun *et al.* 2000; Swanson *et al.* 2001b, 2004; Clark and Swanson 2005; Haerty *et al.* 2007), female reproductive tract function (Lawniczak and Begun 2007; Prokupek *et al.* 2008; Kelleher and Markow 2009), host defense against segregation distorters (Presgraves 2007; Phadnis and Orr 2009), and sperm-egg interactions (Swanson and Vacquier 1995; Swanson *et al.* 2001a; Aagaard *et al.* 2010). Most of these genes are expressed at the latter stages of gametogenesis and often are associated with meiosis or interactions between gametes. However, Civetta *et al.* (2006) and Bauer DuMont *et al.* (2007) independently discovered that two genes expressed in the earliest stages of gametogenesis, specifically germline stem cell (GSC) regulation, also show evidence of adaptive evolution. One of these genes, *bag of marbles* (*bam*), is under intensely strong positive selection with an astonishing 59 nonsynonymous substitutions among 442 codons between two closely related fruit fly species, *Drosophila melanogaster* and *D. simulans* (Civetta *et al.* 2006; Bauer DuMont *et al.* 2007). A second gene, *benign gonial cell neoplasm* (*bgcn*), which acts together with *bam* as a key “switch” to initiate GSC differentiation, is also evolving under positive selection in these two species (Bauer DuMont *et al.* 2007). These discoveries raise a fundamental question: what is the selective pressure(s) driving these adaptive changes at early gametogenesis loci?

There have been several genome-wide, next-generation sequencing surveys of variation in *D. melanogaster* and *D. simulans* that have reported departures from an equilibrium neutral model in directions consistent with natural selection for GSC-related gene ontology categories or at/near several GSC genes (Begun *et al.* 2007; Langley *et al.* 2012; Pool *et al.* 2012). It remains informative to examine specific genes, particularly using parallel assays on population data from both *D. melanogaster* and *D. simulans*. Here, we report high-quality Sanger resequencing from population samples of both species for eight genes involved in GSC regulation (*cyclin A*, *mei-P26*, *nanos*, *P-element induced wimpy testis* (aka *piwi*), *pumilio*, *stonewall*, *fs(1)Yb*, and *zero population growth*), test for evidence of selection using polymorphism-based methods and reanalyze longer-term sequence evolution at these genes using phylogenetic analysis by maximum likelihood (PAML). These eight genes include those whose products genetically and/or physically interact with *bam* and/or *bgcn* and are likely to have shared functions, and those that appear to have non-*bam*/*bgcn*-related roles in GSC regulation. Figure 1 illustrates the roles of these loci within the female germline, wherein the functions and interactions of these genes are more thoroughly understood. We note that several of these genes function somewhat differently in the male germline (Fuller and Spradling 2007; Gilboa and Lehmann 2004; Gonczy *et al.* 1997; Insco *et al.* 2009; Kawase *et al.* 2004; Song *et al.* 2004).

**Figure 1:**
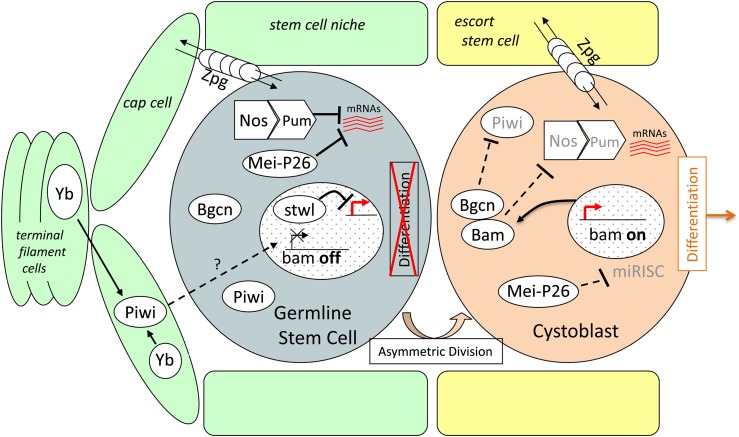
Schematic of the *Drosophila* ovarian germline stem cell (GSC) niche with genes analyzed. Adapted from Wong *et al.* (2005). The GSC (light blue cell) is present in a niche environment (green cells are somatic cap and terminal filament cells, yellow cells are escort stem cells) required to maintain its stem cell state. Bam is repressed in the GSC. Only when the GSC moves away from the niche is Bam expressed and this cell starts to differentiate (tan cell). Yb is involved in the maintenance of GSCs and regulating their division. Piwi acts cell nonautonomously to help in the repression of Bam in the GSC. Zpg is an adherens junction protein that functions in cell signaling. Nos and Pum act as translational repressors of genes that will promote differentiation. Mei-p26 acts in concert with the miRNA machinery (miRISC in the figure) to also repress transcripts (indicated by red squiggly lines), some of which are shared with Nos and Pum. Bgcn is required for Bam to cause GSCs to differentiate. Bam and Bgcn antagonize the Nos/Pum complex. Stwl represses Bam-independent differentiation pathways and thus maintains GSC self-renewal. The cystoblast (tan cell) will undergo four mitotic divisions. CycA participates in the regulation of these mitotic divisions but is not shown in this diagram.

GSCs produce the cells that will develop to form either eggs or sperm throughout a fly’s life. GSCs are maintained in a microenvironment called the stem cell niche that is located in the proximal end of the *Drosophila* ovary or the apical end of the testis (Figure 1). *bam* acts, together with *bgcn*, as a switch to allow for female GSC differentiation, and therefore its expression is repressed in the GSCs (McKearin and Ohlstein 1995; Lavoie *et al.* 1999; Ohlstein *et al.* 2000) by extrinsic signals from the stem cell niche (Song *et al.* 2004). However, this signaling quickly dissipates and thus repression only occurs in cells that are in physical contact with the stem cell niche (Wong *et al.* 2005; Xia *et al.* 2010). To receive these extrinsic signals, GSCs remain physically attached to the niche through adherens junctions (Song *et al.* 2002). The gap junction protein Zero population growth (Zpg) is present in the cytoplasmic membrane of both GSCs and niche cells and is required for the maintenance of GSCs through the sharing of small molecules and signals between the niche and GSC (Tazuke *et al.* 2002; Gilboa *et al.* 2003). Repression of *bam* expression in the GSC is also controlled by the genes *female-sterile(1)Yb* (also abbreviated as *Yb*) and *P-element induced wimpy testis* (*piwi*) (King *et al.* 2001; Szakmary *et al.* 2005).

Intrinsic mechanisms within the GSC play an important role in its maintenance as well, at the levels of transcription and translation. The chromatin-associated protein Stonewall (Stwl) represses genes that promote differentiation (Maines *et al.* 2007), whereas Mei-P26 antagonizes the miRNA pathway and represses transcripts that will promote differentiation in the cystoblast (Neumuller *et al.* 2008; Li *et al.* 2012). At the translational level, Nanos (Nos) and Pumilio (Pum) bind to mRNAs that promote differentiation and inhibit their translation (Lin and Spradling 1997; Wang and Lin 2004). *zpg* is also required to promote cystoblast differentiation (Tazuke *et al.* 2002; Gilboa *et al.* 2003). So depending on the context, *zpg* and *mei-P26* both inhibit and promote GSC differentiation. Finally, the cystoblast will undergo four mitotic divisions. *bam* is thought to regulate the number of mitotic divisions, and genetic interaction assays have suggested that *bam* interacts with the cell cycle factor, *cyclin A* (*cycA*) in this process (Lilly *et al.* 2000).

We report here that all eight genes show a statistically significant departure from an equilibrium neutral model for at least one polymorphism-based statistical test. Additionally, *Yb* and *stwl* also reject neutrality by the McDonald-Kreitman (MK) test, suggesting an excess of nonsynonymous fixations between species consistent with positive selection. These eight genes together with *bam* and *bgcn* have various molecular functions and are expressed in a range of cell types including GSCs, cysts, and surrounding somatic cells suggesting that multiple evolutionary forces are acting throughout the early germline to drive the adaptive evolution of these genes.

## Materials and Methods

### Fly stocks

When possible, African populations of *Drosophila melanogaster* and *D. simulans* were used to minimize the effects of demography in our ability to detect selection (Begun and Aquadro 1993). In some cases, different populations were used for different genes because of the availability of stocks with extracted chromosomes, which allowed us to sequence homozygous lines in *D. melanogaster* for the X, second, or third chromosomes. For *D. simulans* populations, inbred lines were used. For *stwl*, *zpg*, *piwi*, and *pum* a *D. melanogaster* population from Uganda, Africa (Pool and Aquadro 2006) and a *D. simulans* population from Lake Kariba, Zimbabwe, Africa (Pool and Aquadro 2006) were used. For *Yb* and *mei-P26*, a *D. melanogaster* population collected from Sengua Wildlife Research Institute in Zimbabwe, Africa (Begun and Aquadro 1994) and a *D. simulans* population from Lake Kariba, Zimbabwe (Pool and Aquadro 2006) were used. For *cyclin A* and *nanos*, a *D. melanogaster* population sample collected from Lake Kariba, Zimbabwe, Africa (Pool and Aquadro 2006) and an inbred *D. simulans* population sample from North Carolina (Aquadro *et al.* 1988) were used.

### Sequencing

Genomic DNA was extracted from approximately 20 adult flies using Puregene Core Kit A DNA isolation kits (QIAGEN). Polymerase chain reaction and sequencing primer sequences for each gene are listed in Supporting Information, Table S1. Sanger sequencing (both strands) was performed by the Cornell University Genomics Core DNA Sequencing Facility (http://cores.lifesciences.cornell.edu/brcinfo/?f=1) using ABI chemistry and 3730XL DNA Analyzers. Sequences were assembled and edited using Sequencher 4.9 (Gene Codes) and aligned using MEGA 4 (Tamura *et al.* 2007) using default settings, and checked manually to assure the reading frame was retained. Sequences have been deposited in GenBank under accession numbers JX647382-JX647689. For *piwi*, a single 4.8-kb sequence that includes all exons was amplified. This large fragment was problematic for direct sequencing, so it was cloned into the pCR-BluntII-TOPO plasmid (Invitrogen). Two clones of each sample were sequenced to control for PCR errors. If there was ambiguity between the two clones, a third was sequenced and the majority nucleotide was used. The *pum* locus spans over 160 kb, so four separate products were sequenced that include most of the exons (Figure S1A). The *stwl* locus was amplified in two separate products that included both exons (Figure S1B). The *cycA* locus also amplified in two separate products that include two groups of exons in the 5′ and 3′ region of the gene (Figure S1C). For *mei-P26*, only exons 3−6 were amplified. Our results based on this region are consistent with other reports that *mei-P26* has not been subject to recurrent, positive selection (Anderson *et al.* 2009).

### Polymorphism analysis

DnaSP (Librado and Rozas 2009) was used to estimate basic summary statistics of variation within and between species. To detect signatures of recent selection from polymorphism data we applied two quite different tests: OmegaPlus (Pavlidis *et al.* 2010a), which focuses on the linkage disequilibrium signature of selective sweeps, and SweeD (Pavlidis *et al.* 2013), which assesses the fit of the site frequency spectrum to a particular neutral model (it is a faster extension of the widely used SweepFinder method; Nielsen *et al.* 2005).

Statistical significance of OmegaPlus (dependent on linkage disequilibrium) and SweeD (dependent on SFS) test results was determined using neutral simulations with or without demography. We considered a region to be a significant outlier if it fell within the 5% quantile of the simulated datasets. These simulations were done using the program msABC (Pavlidis *et al.* 2010b). We surveyed variation from an African population of *D. melanogaster* which is within this species’ presumed ancestral range. There is mounting evidence that even African populations of this species have experienced changes in effective population size over time (Glinka *et al.* 2003; Haddrill *et al.* 2008; Hutter *et al.* 2007; Li and Stephan. 2006; Duchen *et al.* 2013; Singh *et al.* 2013) and/or migration (Pool *et al.* 2012). Because inferring demographic parameters is challenging, we simulated three different scenarios: standard neutral model with constant population size, standard neutral model with exponential growth as estimated by Hutter *et al.* (2007), or standard neutral model with a 3-phase (“3 epoch”) bottleneck as estimated by Duchen *et al.* (2013). We supplied msABC with uniform prior distributions for theta and all demographic parameters. The theta prior distribution for *D. melanogaster* was obtained from Pool *et al.* (2012) and ranged between 0.006 and 0.009 per site. Figure S2 shows the basic model of the demographic scenarios we considered and the demographic priors used in the simulations. To date, there are no comparable estimates of demographic parameters available for *D. simulans*. Given that the ancestral range of both of these species is in Africa and they are sympatric, we used the *D. melanogaster* demographic parameters as an approximation for *D. simulans*. For *D. simulans*, we used the theta range we observed across the eight GSC loci in this study, which ranged between 0.003 and 0.04 per site.

The MK test (McDonald and Kreitman 1991) was used to test for recurrent, historical positive selection by contrasting pooled polymorphism for *D. melanogaster* and *D. simulans* to fixed differences between species using *D. yakuba* as an outgroup. We used the program DoFE (http://www.sussex.ac.uk/lifesci/eyre-walkerlab/resources) from Eyre-Walker and Keightley (2009) to calculate the proportion of amino acid fixations predicted to be due to positive selection (α). This method uses the site frequency spectrum to jointly estimate the selective effects of new deleterious mutations and the number of adaptive substitution for a selected class of mutations while also incorporating a generalized model of effective population size. For our analysis, we used the site frequency spectrum of fourfold (neutral class) and 0-fold (selected class) codon positions for both *D. melanogaster* and *D. simulans*. The sample size for each locus in our analysis varied for each species. We randomly selected nine and six alleles in *D. melanogaster* and *D. simulans*, respectively. These values correspond to the smallest sample size in each species.

### Divergence analysis

The *Drosophila* 12 Genomes Consortium (2007) has previously reported tests of long-term recurrent positive selection using PAML (Yang 1997, 2007) for *nos*, *pum*, *zpg*, *cycA*, and *mei-P26* and found none departed from a neutral model. Three genes (*stwl*, *piwi*, and *Yb*), had not been included in this previous study due to their strict criteria that ruled out genes with alignment ambiguities. We generated new multiple-sequence alignments using PRANK alignment software (Löytynoja and Goldman 2005) from single sequences of *D. melanogaster*, *D. simulans*, *D. sechellia*, *D. yakuba*, *D. erecta*, and *D. ananassae* downloaded from FlyBase. We did not use more divergent species due to the problems of saturation of synonymous site divergence (The *Drosophila* 12 Genomes Consortium *et al.* 2007). *Yb* from *D. ananassae* has a large number of indels relative to the other five species (and has a much larger coding sequence and an additional intron). Therefore, we analyzed these *Yb* alignments with PAML in two ways: 1) excluding any region with an indel, and 2) excluding any region with an indel as well as with one codon on either side (to reduce the chance calling of “false” substitutions associated with alignment problems). For *Yb*, we also used the recently published improved reference genome sequence of *D. simulans* from Hu *et al.* (2013). The models M0 *vs.* M3, M7 *vs.* M8, and M8 *vs.* M8a were compared. Consistent with the analyses from the *Drosophila* 12 Genomes Consortium (2007), each run was performed using three tree topologies: Tree 1, *D. yakuba* and *D. erecta* as sister species; Tree 2, *D. yakuba* as an outgroup and Tree 3, *D. erecta* as an outgroup. Each model comparison was run under three different initial ω values to assure that convergence was to a global and not local maximum.

#### Adjusting for multiple testing:

We adjusted our criteria for statistical significance by estimating the appropriate *P*-value threshold assuming an experiment-wide Benjamini and Hochberg (1995) false-discovery rate (FDR) of 0.05 using the p.adjust function in the R Project (www.r-project.org). The *P*-values of SweeD and MK tests were combined for correction for each species separately as both tests use the frequency or counts of each polymorphism. OmegaPlus only uses patterns of linkage disequilibrium across sites, and thus those *P*-values were corrected separately (again for each species alone).

## Results

### Polymorphism-based analyses

Gene function and sample size data from African populations of *D. melanogaster* and either African or North American *D. simulans* are reported in Table 1, and standard summary statistics for each gene in Table 2. We find that *D. simulans* levels of nucleotide variability are generally higher than those seen in *D. melanogaster*, consistent with previous results (Aquadro *et al.* 1988).

**Table 1:** Genes surveyed and sample sizes

Gene	Function	Number of Alleles Sampled	
		*D. melanogaster*	*D. simulans*
*cyc A**^a^*	Regulation of cyst mitotic divisions		
Segment 1		9	10
Segment 2		9	10
*Yb*	GSC maintenance and cytoblast differentiation	19	9
*mei-P26**^a^*	GSC maintenance	19	10
*nos**^a^*	GSC maintenance	9	10
*pum**^a^*	GSC maintenance		
Segment 1		17	9
Segment 2		11	10
Segment 3		19	9
Segment 4		18	7
*piwi*	GSC maintenance	10	6
*stwl*	Chromatin factor, GSC maintenance		
Segment 1		18	8
Segment 2		15	9
*zpg**^a^*	GSC adherens junction and cystoblast differentiation	18	10

a Indicates that gene has a genetic and/or physical interaction reported with *bam*. For *pumilio*, four separate regions were amplified and analyzed, labeled as 1-4. For *stonewall* and *cycA* two separate regions were amplified, labeled as 1 and 2. GSC, germline stem cell.

**Table 2:** Nucleotide polymorphism estimates for GSC genes

Gene	Species	S	θ	π_Tot_	π_Syn_	π_Non_
*cycA 1*	*D. melanogaster*	14	0.0051	0.0046	0.0025	0.0054
	*D. simulans*	10	0.0035	0.0044	0.0277	0.0000
*cycA 2*	*D. melanogaster*	15	0.0085	0.0074	0.0157	0.0010
	*D. simulans*	11	0.0056	0.0061	0.0164	0.0000
*mei-P26*	*D. melanogaster*	26	0.0062	0.0061	0.0181	0.0000
	*D. simulans*	21	0.0055	0.0031	0.0110	0.0000
*nos*	*D. melanogaster*	21	0.0044	0.0045	0.0090	0.0009
	*D. simulans*	35	0.0097	0.0093	0.0150	0.0042
*piwi*	*D. melanogaster*	103	0.0079	0.0074	0.0196	0.0024
	*D. simulans*	196	0.0222	0.0204	0.0368	0.0025
*pumilio 1*	*D. melanogaster*	26	0.0040	0.0046	0.0033	0.0012
	*D. simulans*	103	0.0202	0.0169	0.0142	0.0003
*pumilio 2*	*D. melanogaster*	10	0.0052	0.0040	0.0072	0.0000
	*D. simulans*	33	0.0142	0.0144	0.0346	0.0005
*pumilio 3*	*D. melanogaster*	10	0.0040	0.0046	0.0172	0.0020
	*D. simulans*	72	0.0400	0.0388	0.0685	0.0014
*pumilio 4*	*D. melanogaster*	13	0.0021	0.0020	0.0070	0.000
	*D. simulans*	50	0.0095	0.0089	0.0207	0.0011
*stwl 1*	*D. melanogaster*	21	0.0092	0.0058	0.0000	0.0001
	*D. simulans*	17	0.0088	0.0064	0.0119	0.0000
*stwl 2*	*D. melanogaster*	49	0.0051	0.0048	0.0123	0.0025
	*D. simulans*	43	0.0053	0.0050	0.0097	0.0033
*Yb*	*D. melanogaster*	88	0.0079	0.0060	0.0129	0.0028
	*D. simulans*	111	0.0128	0.0128	0.0259	0.0085
*zpg*	*D. melanogaster*	41	0.0095	0.0113	0.0413	0.0001
	*D. simulans*	60	0.0164	0.0148	0.0286	0.0015

Each amplified region of *cycA*, *pumilio*, and *stwl* was analyzed separately; see the section *Materials and Methods* and Figure S1 for locations of each amplicon. S, segregating sites; θ, nucleotide diversity; π_Tot_, total diversity; π_syn_, synonymous diversity; π_non_, nonsynonymous diversity. GSC, germline stem cell.

Analysis of the polymorphism site frequency data using SweeD reveals significant departures from neutrality at 15 of 16 gene/species comparisons after multiple-testing correction (Table 3). For this tabulation, we consider a gene to be showing a significant departure from neutrality if at least one of the gene regions analyzed shows a significant departure (after multiple test correction) for all three demographic scenarios (standard neutral, exponential growth, and 3-epoch bottleneck). Only *piwi* in *D. simulans* fits a neutral model under all three demographic scenarios.

**Table 3:** Site frequency tests of departures from neutral models for eight GSC genes in *D. melanogaster* and *D. simulans*

Test Details	SweeD Test of Recent Selection				OmegaPlus Test of Recent Selection			
	*D. melanogaster*		*D. simulans*		*D. melanogaster*		*D. simulans*	
	Orig *P*-value	FDR adj *P*-Value	Orig *P*-Value	FDR adj *P*-Value	Orig *P*-Value	FDR adj *P*-Value	Orig *P*-Value	FDR adj *P*-Value
CycA1.SN	0.0007	**0.0023**	0.0015	**0.0032**	0.367	0.4037	0.4756	0.4756
CycA1.Ex	0.0007	**0.0023**	0.0009	**0.0023**	0.4323	0.4390	0.4049	0.4711
CycA1.3Ep	0.0007	**0.0023**	0.0012	**0.0027**	0.439	0.4390	0.4094	0.4711
CycA2.SN	0.6203	0.7030	0.9933	0.9933	0.3726	0.4037	0.2594	0.4214
CycA2.Ex	0.3460	0.4334	0.7363	0.8024	0.2809	0.3894	0.2622	0.4214
CycA2.3Ep	0.3467	0.4334	0.7863	0.8460	0.2866	0.3894	0.2595	0.4214
meiP26.SN	0.0010	**0.0023**	0.0009	**0.0023**	0.1894	0.3894	0.0333	0.4214
meiP26.Ex	0.0008	**0.0023**	0.0010	**0.0023**	0.2081	0.3894	0.0999	0.4214
meiP26.3Ep	0.0008	**0.0023**	0.0006	**0.0023**	0.2108	0.3894	0.0648	0.4214
nano.SN	0.0090	**0.0150**	0.0008	**0.0023**	0.4055	0.4274	0.2278	0.4214
nano.Ex	0.0020	**0.0040**	0.0010	**0.0023**	0.2560	0.3894	0.2329	0.4214
nano.3Ep	0.0050	**0.0092**	0.0009	**0.0023**	0.3403	0.4037	0.2204	0.4214
piwi.SN	0.0009	**0.0023**	0.9680	0.9795	0.1267	0.3894	0.1899	0.4214
piwi.Ex	0.0070	**0.0124**	0.9370	0.9713	0.1096	0.3894	0.3899	0.4711
piwi.3Ep	0.0010	**0.0023**	0.9500	0.9729	0.1030	0.3894	0.3909	0.4711
pum1.SN	0.0008	**0.0023**	0.4174	0.4997	0.2302	0.3894	0.2198	0.4214
pum1.Ex	0.0009	**0.0023**	0.1640	0.2213	0.2537	0.3894	0.2126	0.4214
pum1.3Ep	0.0009	**0.0023**	0.1736	0.2306	0.2532	0.3894	0.2293	0.4214
pum2.SN	0.0008	**0.0023**	0.0919	0.1281	0.3631	0.4037	0.2649	0.4214
pum2.Ex	0.0009	**0.0023**	0.0278	**0.0407**	0.3630	0.4037	0.2599	0.4214
pum2.3Ep	0.0008	**0.0023**	0.0227	**0.0339**	0.3389	0.4037	0.2605	0.4214
pum3.SN	0.2073	0.2711	0.0153	**0.0245**	0.1728	0.3894	0.1664	0.4214
pum3.Ex	0.1633	0.2213	0.0010	**0.0023**	0.0612	0.3894	0.2701	0.4214
pum3.3Ep	0.0800	0.1133	0.0019	**0.0039**	0.0800	0.3894	0.2131	0.4214
pum4.SN	0.0186	**0.0287**	0.8249	0.8765	0.2995	0.3894	0.4296	0.4711
pum4.Ex	0.0067	**0.0121**	0.7174	0.7919	0.2694	0.3894	0.3909	0.4711
pum4.3Ep	0.0080	**0.0139**	0.6710	0.7505	0.2690	0.3894	0.3414	0.4711
stwlReg1.SN	0.0159	**0.0250**	0.0219	**0.0332**	0.0885	0.3894	0.0444	0.4214
stwlReg1.Ex	0.0009	**0.0023**	0.0021	**0.0042**	0.0630	0.3894	0.1062	0.4214
stwlReg1.3Ep	0.0043	**0.0081**	0.0010	**0.0023**	0.0516	0.3894	0.0827	0.4214
stwlReg2.SN	0.0010	**0.0023**	0.8780	0.9214	0.2987	0.3894	0.4099	0.4711
stwlReg2.Ex	0.0006	**0.0023**	0.4943	0.5756	0.2851	0.3894	0.4637	0.4756
stwlReg2.3Ep	0.0007	**0.0023**	0.4688	0.5534	0.2869	0.3894	0.4349	0.4711
yb.SN	0.0009	**0.0023**	0.0014	**0.0031**	0.0009	**0.0351**	0.4469	0.4711
yb.Ex	0.0006	**0.0023**	0.0040	**0.0077**	0.0059	0.0767	0.4212	0.4711
yb.3Ep	0.0008	**0.0023**	0.0007	**0.0023**	0.0033	0.0644	0.3702	0.4711
zpg.SN	0.0008	**0.0023**	0.0009	**0.0023**	0.2129	0.3894	0.2093	0.4214
zpg.Ex	0.0090	**0.0150**	0.0006	**0.0023**	0.2991	0.3894	0.1461	0.4214
zpg.3Ep	0.0010	**0.0023**	0.0009	**0.0023**	0.2807	0.3894	0.1692	0.4214

Simulations to establish *P*-values were from the standard neutral model (SN), exponential growth model (Ex), or a 3-epoch model (3Ep; large, small, large population size) as described in the section *Materials and Methods*. FDR-adjusted *P*-values were determined as described in text. Significant results (*P* < 0.05) are in bold. FDR, false-discovery rate.

OmegaPlus rejected the standard neutral model only for *Yb* in *D. melanogaster* after multiple test correction at the 0.05 FDR level (Table 3). The generally short size of the regions analyzed may have limited the statistical power of the OmegaPlus method, which relies on a unique structure of linkage disequilibrium generated by recent selective sweeps.

### Polymorphism and divergence-based tests

The McDonald-Kreitman (MK) test rejected neutrality for both *Yb* and *stwl* after correction for multiple testing (Table 4). The method of Bauer DuMont *et al.* (2004) suggests that these MK test rejections are not due to selection on synonymous sites for either gene. High d_N_/d_S_ ratios between species (0.627 for *Yb*, and 0.502 for *stwl*) compared with the genome-wide average of 0.0125 (Larracuente *et al.* 2008), yet normal levels of d_S_ for both genes (0.132 and 0.119, respectively), suggests that the MK test rejections are due to excesses of fixed nonsynonymous differences between species consistent with positive selection.

**Table 4:** MK tests of departures from a neutral model for eight GSC genes using polymorphism within both *D. melanogaster* and *D. simulans* and fixed differences between species

Gene	Synon Poly	Synon Div	Nonsyn Poly	Nonsyn Div	*P*-Value	FDR adj *P*-Value
*cyc A*	17	14	4	6	0.414	0.4997
*mei-P26*	24	44	0	0	NA	NA
*nos*	11	11	7	23	0.046	0.0663
*piwi*	84	54	26	22	0.416	0.4997
*pum*	77	51	11	5	0.506	0.5812
*stwl*	45	62	48	124	0.015	**0.0245**
*Yb*	86	62	80	149	0.00001	**0.0010**
*zpg*	53	14	6	4	0.230	0.2962

FDR adjusted *P*-values were determined as described in text. Significant results after FDR (*P* < 0.05) are in bold. MK, McDonald-Kreitman; GSC, germline stem cell; FDR, false-discovery rate.

Using the DoFE program of Eyre-Walker and Keightley (2009), we estimated, in both *D. melanogaster* and *D. simulans*, the overall proportion of amino acid substitutions fixed due to positive selection (α), and the 95% credibility interval around this estimate (supplemental method presented in Eyre-Walker and Keightley 2009). This analysis uses the site frequency spectrum across the eight GSC loci to estimate the distribution of fitness effects acting on new deleterious mutations, while incorporating a general model of effective population size change. The distribution of fitness effects is then used to determine the proportion of amino acid fixations that are due to positive selection. For the eight loci in our study, we estimate α to be 0.814 (95% credibility interval: 0.698−0.896) and 0.790 (95% credibility interval: 0.681−0.881) for *D. melanogaster* and *D. simulans*, respectively. We also analyzed the X and autosomal loci separately. For *D. melanogaster* we observe a α of 0.934 (95% credibility interval: 0.852−0.979) for the X chromosome and 0.672 (95% credibility interval: 0.413−0.836) for the autosomes. For *D. simulans* we observe a α of 0.856 (95% credibility interval: 0.695−0.957) for the X chromosome and 0.743 (95% credibility interval: 0.579−0.876) for the autosomes. The autosomal 95% credibility interval estimated for α from our *D. melanogaster* data encompasses the α estimate obtained from sequence data from 419 autosomal loci chosen randomly (0.52; Keightley and Eyre-Walker 2012). To date, this method to estimate α has not been applied to another *D. simulans* dataset. However, α has been calculated by other methods for *D. simulans* and estimates have ranged from 0.43 to 0.94 (reviewed in Eyre-Walker 2006), which is similar to the estimates we present here.

### Divergence-based analyses

No evidence of recurrent, adaptive evolution at the same subset of codons across *D. melanogaster*, *D. simulans*, *D. sechellia*, *D. yakuba*, *D. erecta*, and *D. ananassae* was detected using PAML (Yang 1997, 2007) for seven of the eight genes *cycA*, *mei-P26*, *nos*, *piwi*, *pum*, *stwl*, *zpg* (our analyses and those presented in The *Drosophila* 12 Genomes Consortium 2007). However, we do find evidence of recurrent, positive selection at specific codons for *Yb*. Using both models M7 *vs.* M8, and M8 *vs.* M8a, we find that the data fit a model of selection significantly better than a neutral null model (likelihood ratio test statistics of 16.068 with *P* < 0.0003, and 6.321 with *P* < 0.01, respectively). This result is robust to alignment with this highly diverged protein, including reanalysis removing all codons adjacent to predicted INDELS. 19 of the aligned codons at *Yb* are predicted by Bayes Empirical Bayes analysis to be in the selective class with an average codon-specific d_N_/d_S_ (= ω) of 1.88. However, only two codons in this class have predicted posterior probabilities greater than 0.90, and they do not fall in areas of known domains.

## Discussion

Previous genome-wide next-generation sequencing studies using both site frequency-based and MK tests of neutrality have reported an enrichment of putative adaptive evolution in Gene Ontology categories such as germ-cell development, cystoblast division, and germarium-derived oocyte fate determination (Begun *et al.* 2007; Langley *et al.* 2012; Mackay *et al.* 2012). In this study, we performed high-quality Sanger sequencing of population samples from both *D. melanogaster* and *D. simulans* and found that all eight genes involved in GSC regulation studied here reject a neutral model of evolution in at least one test and species (Tables 3 and 4). Most of these rejections are due to the polymorphism-based SweeD analysis for which every locus, except *piwi*, rejects the neutral model in both *D. melanogaster* and *D. simulans*. The *piwi* locus only rejects neutrality by the SweeD test in *D. melanogaster*. Rejecting the neutral model with SweeD is suggestive of positive selection, but it could also be due to demographic history (Pavlidis *et al.* 2010a). We attempted to take the demographic history of these species into account by using simulated replicates of estimates of *D. melanogaster* African population dynamics (Hutter *et al.* 2007; Duchen *et al.* 2013) to determine our significant SweeD cutoff points. However, the true demographic history of these species is unknown. So, we stress that our SweeD rejections are restricted to the demographic scenarios we considered.

The detection of outliers of a test statistic’s genomic distribution is another method used to determine statistical significance. Recently Pool *et al.* (2012) applied SweeD (labeled SweepFinder in their manuscript) genome-wide for an African population of *D. melanogaster* and they list regions containing genomic outliers, assumed to be due to positive selection. As an attempt to determine if our SweeD rejections are more likely due to demography *vs.* selection, we checked to see if the eight GSC loci we analyzed fell within or near the Pool *et al.* (2012) outliers. The protein coding regions (CDS) for three GSC loci (*Yb*, *piwi* and *mei-P26*) are within an outlier region, suggesting that for these loci our SweeD rejections are due to positive selection. The CDS for two other GSC loci (*zpg* and *nano*) are within 50 kb of an outlier region. Simulations have shown that SweeD’s ability to pinpoint the target of selection is compromised if both selection and demographic perturbations have occurred (Pavlidis *et al.* 2010a) with the predicted target being tens of kilobases away from the actual location of selection. To determine if by chance one would expect to observe three of eight loci within an outlier regions, or five of eight loci 50 kb from an outlier region, we randomly picked eight loci from the *D. melanogaster* genome. The loci were picked such that we obtained a random sample with the same distribution across the X, 2^nd^, or 3rd chromosomes as observed across the GSC loci. For both cases our observation is significant with only 36 of 1000 bootstrapped samples having 3 and greater or 5 and greater loci within or 50 Kb from an outlier region, respectively (thus *P*-value = 0.036 for our observation). Therefore, for 5 of the 8 GSC loci we analyzed, two different datasets (using two different methods for determining the significant cutoffs) suggest that their frequency spectra do not match the neutral model in *D. melanogaster*. For *D. simulans*, making a distinction between demography and selection is more tenuous, especially given that there are no comparable estimates of the demographic history within Africa for this species.

*Yb* is the only gene to show significant departures from neutrality consistent with natural selection for the site frequency test SweeD as well as for both the MK and PAML tests that can detect recurrent historical selection. This combination of test results suggests that the recent sweeps at *Yb* detected by SweeD are just the latest of many selective fixations of nonsynonymous substitutions that have occurred among these six species.

Using the method of Eyre-Walker and Keightley (2009), we estimate that 81% of the amino acid differences fixed in these eight genes in the *D. melanogaster* lineage and 79% of the amino acid differences fixed in *D. simulans* lineage have been driven by positive selection. Estimates for X-linked genes were slightly, although not significantly, larger than those for autosomes. This proportion is on the upper end of that estimated for other groups of genes in these species.

The pattern of evidence for recent or recurrent positive selection that we observe and the diverse functions and expression patterns of these genes suggest that there are likely multiple selective pressures driving the adaptive evolution in genes important in GSC regulation. For example, three of the eight genes examined that reject the neutral model have no known interaction or dependence on *bam* function (*stwl*, *piwi*, and *Yb*: Chen and McKearin 2005; Li *et al.* 2009). *Yb* is expressed in the stem cell niche (King and Lin 1999; King *et al.* 2001), whereas *stwl* binds chromatin (Clark and McKearin 1996; Maines *et al.* 2007), making it less likely that the same specific selective pressures act on both.

The hypothesis of sexual selection and sexual conflict (Swanson and Vacquier 2002) cannot be formally rejected but seem implausible for genes functioning in GSCs. For example, most theories of sexual selection predict strong effects on premating traits, which are highly unlikely to be influenced by the genes we have examined. Likewise, sexual conflict, whereby one sex manipulates the reproductive fitness of the other sex, is much more likely to occur for molecules that are transmitted between males and females, a function that is implausible for any of the GSC regulatory genes in this study.

Several other mechanistic and evolutionary hypotheses have been proposed to explain the evolutionary causes of positive selection inferred for *bam* and *bgcn*. Some of these selective pressures also may drive the adaptive evolution of other genes involved in GSC regulation. Civetta *et al.* (2006) proposed that species-specific changes in rates of proteolysis could drive protein sequence divergence. This proposal was supported by the observation that *bam*’s expression is transient and by previous studies in *C. elegans* that have shown that transiently expressed genes have elevated rates of protein evolution (Cutter and Ward 2005). Although this could influence the molecular evolution of *bam*, and potentially *bgcn* which is also transiently expressed (Ohlstein *et al.* 2000), it is unlikely to explain all selection acting on GSC gene evolution since *piwi*, *Yb*, *stwl*, and *nanos* have much broader patterns and timings of expression (Clark and McKearin 1996; Forbes and Lehmann 1998; Cox *et al.* 2000; Szakmary *et al.* 2009).

We had previously hypothesized that coevolution with external pathogens infecting the germline could underlie the elevated nonsynonymous divergence in *bam* and *bgcn* along the *D. melanogaster* and *D. simulans* lineages (Bauer DuMont *et al.* 2007). Two maternally-inherited bacterial endosymbionts (*Wolbachia* and *Spiroplasma*) have been detected in some but not all species of Drosophila (Mateos *et al.* 2006; Watts *et al.* 2009). Infection by *Wolbachia* can have beneficial effects in some species by increasing resistance to viral infections, which may explain their widespread presence (Chrostek *et al.* 2013; Hedges *et al.* 2008; Teixeira *et al.* 2008). However, *Wolbachia* infection can also reduce fecundity due to cytoplasmic incompatibilities in crosses between infected and uninfected individuals (Fry *et al.* 2004). Overreplication of *Wolbachia* also has been linked to shortening life-span and rupture of host cells (Min and Benzer 1997). There is likely to be a delicate balance in controlling endosymbiont proliferation within a cell so that the host can receive benefits from the endosymbiont but minimize any deleterious effects (Chrostek *et al.* 2013). Maintaining such a balance could contribute to an “arms race” between GSC regulatory genes and endosymbionts (*e.g.*, Werren 2005; Bauer DuMont *et al.* 2007).

The expression patterns and known pleiotropic functions of *Yb*, *piwi*, *stwl* (Aravin *et al.* 2007; Brennecke *et al.* 2008; Clark and McKearin. 1996; Maines *et al.* 2007) suggest that other pressures may be acting on them. One possible selective pressure is intracellular parasites such as transposons. Transposons are selfish genetic elements that can propagate throughout the genome, resulting in deleterious effects on their host. Recent studies demonstrated that many taxa, including *Drosophila*, have a small RNA silencing pathway, termed the piRNA pathway, that is active in the germline and provides an adaptive defense against transposons (Aravin *et al.* 2007). Many piRNA pathway genes also have been shown to adaptively evolve (Obbard *et al.* 2009; Kolaczkowski *et al.* 2011). *piwi* and *Yb* are required for the proper silencing of transposons (Aravin *et al.* 2007; Olivieri *et al.* 2010; Saito *et al.* 2010). Therefore, the adaptive evolution seen in these two proteins may reflect their involvement in silencing transposons as previously suggested for *piwi* (Obbard *et al.* 2009; Kolaczkowski *et al.* 2011).

Additionally, it is possible that selective pressure to repress transposons may be driving the adaptive evolution of *stwl* since some other chromatin-associated proteins are involved in transposon silencing (Klattenhoff *et al.* 2009; Rangan *et al.* 2011). Species-specific changes in life history and the timing of reproduction could also pose changing selective pressures on the germline (Schmidt and Paaby 2008), though our limited knowledge of the ages of reproduction for natural populations of *Drosophila* limits our ability to test this hypothesis.

In the future, it will be important to test whether these positively selected GSC genes function in the specific biological processes that we hypothesize are driving their adaptive evolution. For example, do *bam* or *bgcn* play a role in regulating the transmission of bacterial endosymbionts, or does *stonewall* act in the repression of transposons? Additional insight may come from sampling these genes from additional *Drosophila* species to determine whether they have experienced a long-term selective pressure across many *Drosophila* or whether it is specific to *D. melanogaster* and *D. simulans*.

## 

## Supplementary Material

Supporting Information
