# Supplementary material for: Adaptive Evolution of Genes Involved in the Regulation of Germline Stem Cells in Drosophila melanogaster and D. simulans
Source: G3 (Bethesda). 2015 Feb 9;5(4):583–92. doi: 10.1534/g3.114.015875 (PMC4390574; doi:10.1534/g3.114.015875)
Supplement: Supporting Information [file supp_5_4_583__index.html]

Adaptive Evolution of Genes Involved in the Regulation of Germline Stem Cells in Drosophila melanogaster and D. simulans — Supporting Information 

# Adaptive Evolution of Genes Involved in the Regulation of Germline Stem Cells in *Drosophila melanogaster* and *D. simulans*

## Supporting Information for Flores *et al.*, 2015

**Files in this Data Supplement:**

- Supporting Information - Figures S1-S2 and Table S1 (PDF, 257 KB)
- Figure S1 - Sequenced fragments of *pumilio*, *stonewall*, and *cyclin A*. (PDF, 189 KB)
- Figure S2 - Basic models of the demographic scenarios we considered and the demographic priors used in the simulations to evaluate statistical significance of OmegaPlus and SweeD test results. (PDF, 144 KB)
- Table S1 - Primers used in this study. (PDF, 113 KB)
