# Supplementary material for: Adaptive Evolution of Genes Involved in the Regulation of Germline Stem Cells in Drosophila melanogaster and D. simulans
Source: G3 (Bethesda). 2015 Feb 9;5(4):583–92. doi: 10.1534/g3.114.015875 (PMC4390574; doi:10.1534/g3.114.015875)
Supplement: Supporting Information [file supp_g3.114.015875_015875SI.pdf]

G3: Genes | Genomes | Genetics

Adaptive evolution of genes involved in the regulation of germline stem cells in *Drosophila melanogaster* and *D. simulans*

Heather A. Flores, Vanessa L. Bauer DuMont, Aalya Fatoo, Diana Hubbard, and Mohammed Hijji, Daniel A. Barbash, Charles F. Aquadro[1]

Molecular Biology & Genetics, Cornell University, Ithaca, NY 14853, USA

[1]Corresponding Author: Department of Molecular Biology and Genetics, Biotechnology Building, Cornell University, Ithaca, NY 14853 Phone: 607-254-4838

Genbank accession numbers: JX647382-JX647689

DOI: 10.1534/g3.114.015875

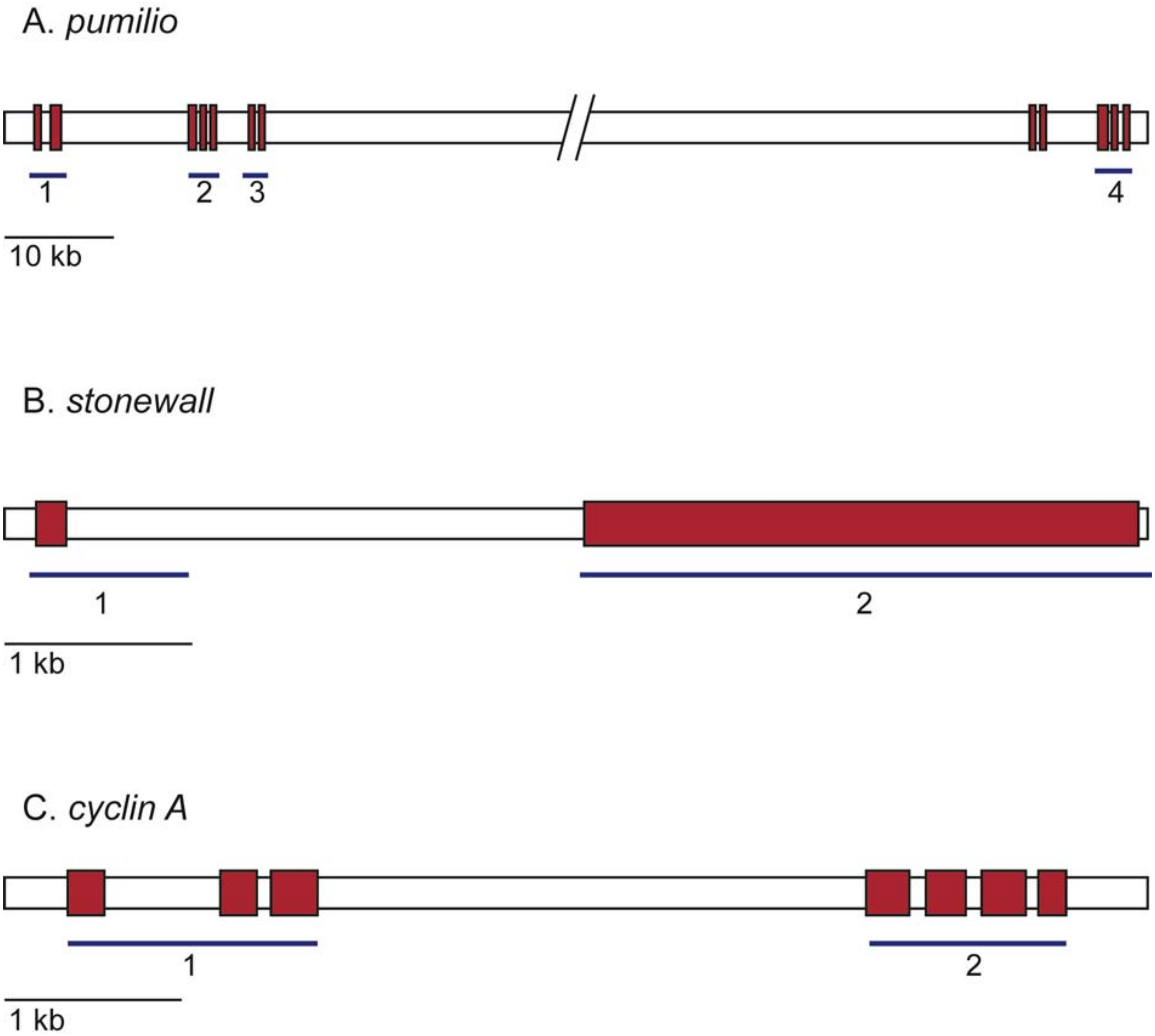

Figure S1 Sequenced fragments of *pumilio*, *stonewall*, and *cyclin A.* (A) *pum* sequencing. The *pum* locus spans approximately 160 kb, so four different regions of *pum*, labeled 1-4, that include 10 of 12 exons in were individually sequenced. The diagram corresponds to the *pum*-A isoform. The center hashes denote where internal sequence (~70 kb) was removed to allow for ease of viewing. (B) *stwl* sequencing. Two fragments of *stwl* were amplified, labeled 1-2. (C) *cycA* sequencing. Two fragments of *cycA* were amplified, labeled 1-2. The blue lines denote the amplified fragments. Red boxes denote exons.

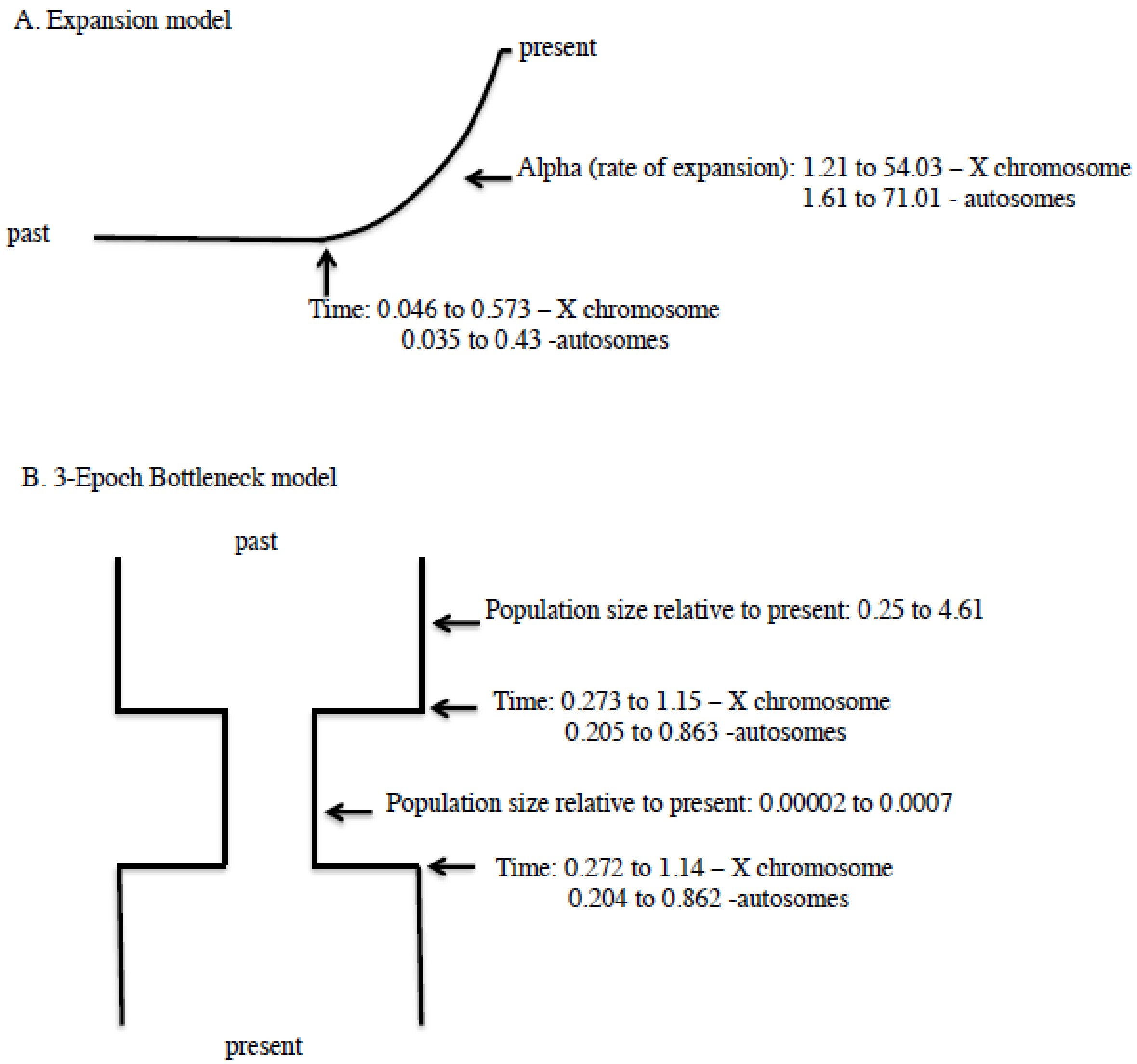

Figure S2 Basic models of the demographic scenarios we considered and the demographic priors used in the simulations to evaluate statistical significance of OmegaPlus and SweeD test results. Time is in units of $3N_e$ and $4N_e$ generations for the X chromosome and autosomes, respectively. N_e is the effective population, which we assume to be approximately 1 x 10^6 for *D. melanogaster* and *D. simulans*. We also assume 10 generations per year for these species. We note that the distribution of the two bottleneck Time parameters overlap in the 3-Epoch model. Therefore, for the 3-Epoch model, msABC was set-up such that we only obtained simulation replicates from draws of the Time distributions where the ordering were correct (i.e., the most current Time value is smaller than the Time farther in the past).

Table S1 Primers used in this study

Gene	Species	Name	Sequence
cyclin A (1)	mel/sim	cycaF1	CAGTTTCCAGATCCACCAAG
cyclin A (1)	mel/sim	cycaR1	TTTAGCTTACCTCGCTCTCC
cyclin A (2)	mel/sim	cycaF2	TCTTCCAGAAGAAACATCGC
cyclin A (2)	mel/sim	cycaR2	GTATTAATATCCGGCTGCTG
nanos	mel/sim	nosF1	CAGCAACTTGGAGGGCAGTG
nanos	mel/sim	nosR1	AAACCTTCATCTGTTGCTTG
fs(1)Yb	mel	yb_sim_F1	CCTCGCTAGCCGTACATATATTAG
fs(1)Yb	mel	yb_sim_R1	GGTCAGTGGACAGTGATGAAAC
mei-P26	mel/sim	mei-p26_F2	GATGGGCTTTTGTTGAACGG
mei-P26	mel/sim	mei-p26_R2	TGCTGTTGCAGATGGTGTTG
piwi	mel/sim	piwi_F1	TTCAAAGTACTCTTTCAGTTTCC
piwi	mel/sim	piwi_R1	GTCTGGGCTAGTTTCATATATGG
pumilio (1)	mel/sim	pum_F1	CCCTACTTTCAACAGCTACAC
pumilio (1)	mel	pum_R1	CAAGCCAAGAAAGTTAACC
pumilio (1)	sim	pum_sim_R1	CAAGCCAAGAAAATTAACC
pumilio (2)	mel/sim	pum_F3	GATATTTGCTTTCCTGGAAGCC
pumilio (2)	mel/sim	pum_R3	GTCTGGGGTCTTTAGTCGG
pumilio (3)	mel	pum_F4	GGCTAAGTGGTGAATACAG
pumilio (3)	sim	pum_sim_F4	AACGTTTTAATGATAGCTTG
pumilio (3)	mel	pum_R4	GAAAATGTCACTCTGGGGAC
pumilio (3)	sim	pum_sim_R4	GAAAATGTCACTCTGGAGAC
pumilio (4)	mel	pum_F8	CATTCTCCTCTATACCTTTCC

pumilio (4)	sim	pum_sim_F8	CATTCTCTTTGATACCTCTCC
pumilio (4)	mel/sim	pum_R8	GAAGTTTCCTTTGACTGCCTG
stonewall (1)	mel/sim	stwl_F1_1	GATTGTGTGAATTGCGTTTG
stonewall (1)	mel/sim	stwl_R1_1	CTAATGGGCGATTAGTGTTAC
stonewall (2)	mel/sim	stwl_F2	CTAGCCTTATCATTTCCCTC
stonewall (2)	mel/sim	stwl_R2	CTCTTTAATCAATACTCGG
zpg	mel	zpg_F1	GTCAAACTTTACAACCGCC
zpg	sim	zpg_sim_F1	GTCAAACTTTACAAGCACC
zpg	mel	zpg_R1	GATTAAACTTGGCGTCATC
zpg	sim	zpg_sim_R1	GATTAAACTTGGTGTCATC
