# Supplementary material for: Adaptive Evolution of Genes Involved in the Regulation of Germline Stem Cells in Drosophila melanogaster and D. simulans
Source: G3 (Bethesda). 2015 Feb 9;5(4):583–92. doi: 10.1534/g3.114.015875 (PMC4390574; doi:10.1534/g3.114.015875)
Supplement: Supporting Information [file supp_g3.114.015875_FigureS1.pdf]

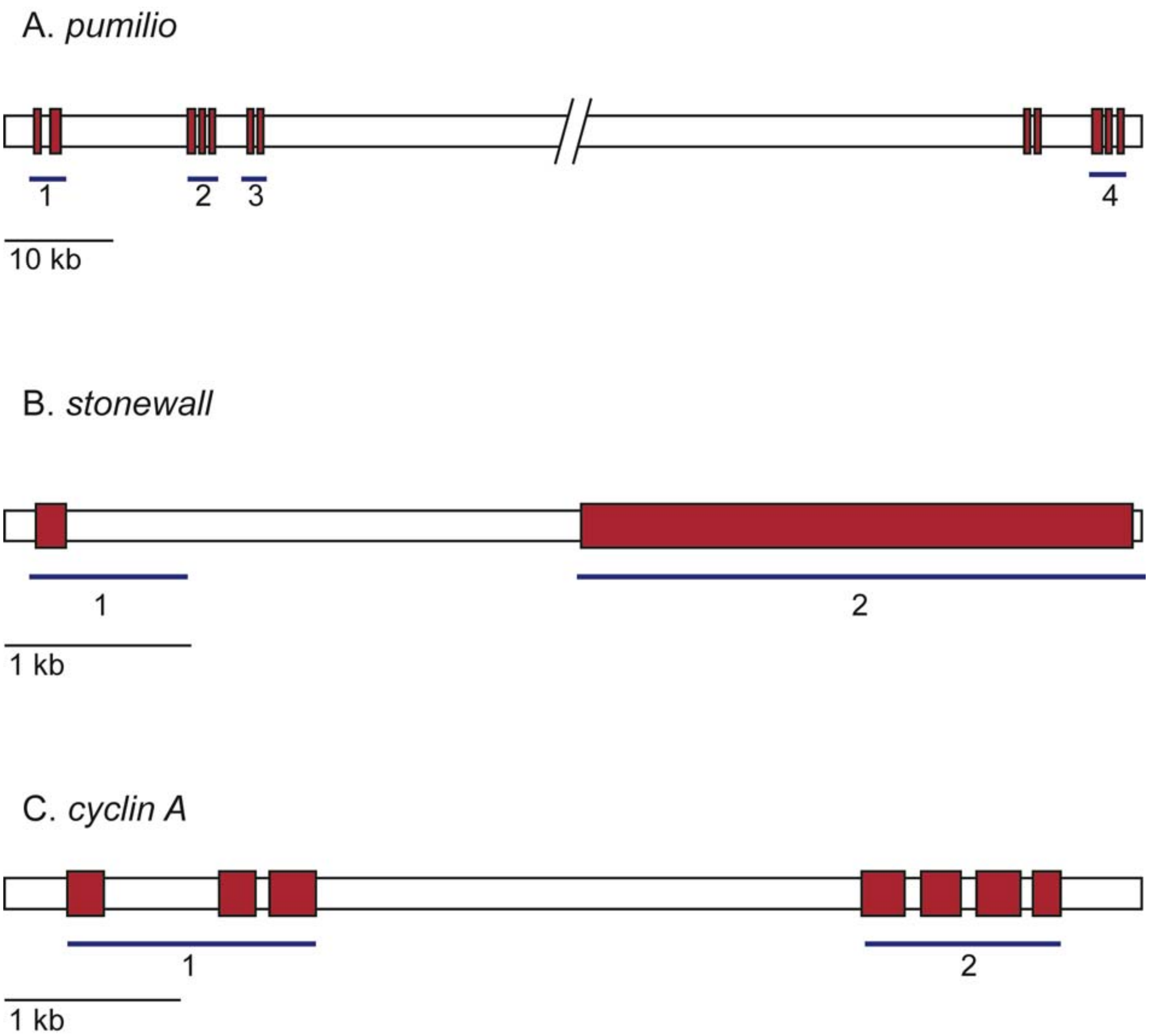

Figure S1 Sequenced fragments of *pumilio*, *stonewall*, and *cyclin A.* (A) *pum* sequencing. The *pum* locus spans approximately 160 kb, so four different regions of *pum*, labeled 1-4, that include 10 of 12 exons in were individually sequenced. The diagram corresponds to the *pum*-A isoform. The center hashes denote where internal sequence (~70 kb) was removed to allow for ease of viewing. (B) *stwl* sequencing. Two fragments of *stwl* were amplified, labeled 1-2. (C) *cycA* sequencing. Two fragments of *cycA* were amplified, labeled 1-2. The blue lines denote the amplified fragments. Red boxes denote exons.
