# Supplementary material for: Adaptive Evolution of Genes Involved in the Regulation of Germline Stem Cells in Drosophila melanogaster and D. simulans
Source: G3 (Bethesda). 2015 Feb 9;5(4):583–92. doi: 10.1534/g3.114.015875 (PMC4390574; doi:10.1534/g3.114.015875)
Supplement: Supporting Information [file supp_g3.114.015875_FigureS2.pdf]

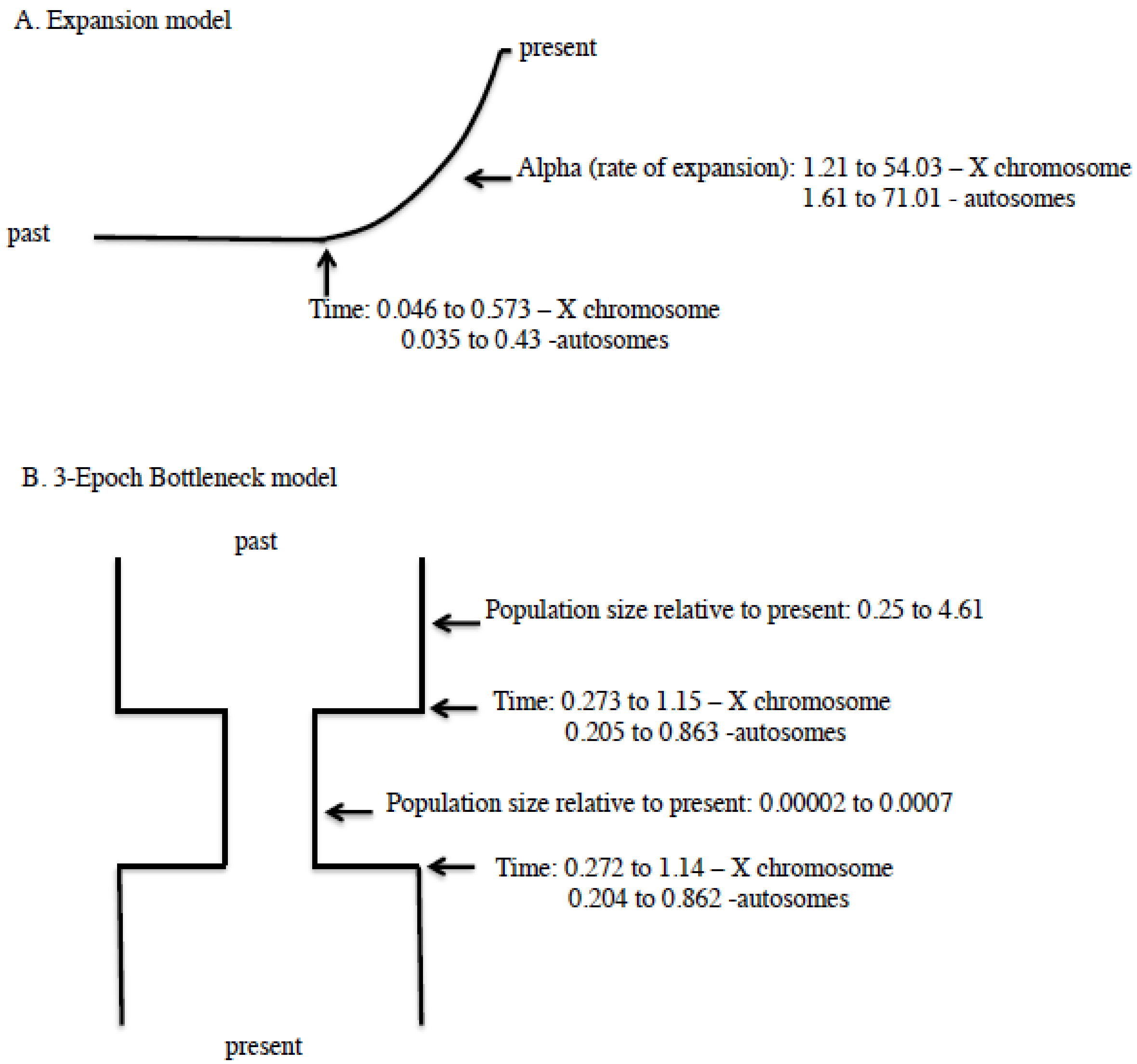

Figure S2 Basic models of the demographic scenarios we considered and the demographic priors used in the simulations to evaluate statistical significance of OmegaPlus and SweeD test results. Time is in units of $3N_e$ and $4N_e$ generations for the X chromosome and autosomes, respectively. N_e is the effective population, which we assume to be approximately 1 x 10^6 for *D. melanogaster* and *D. simulans*. We also assume 10 generations per year for these species. We note that the distribution of the two bottleneck Time parameters overlap in the 3-Epoch model. Therefore, for the 3-Epoch model, msABC was set-up such that we only obtained simulation replicates from draws of the Time distributions where the ordering were correct (i.e., the most current Time value is smaller than the Time farther in the past).
