# Supplementary material for: Adaptive Evolution of Genes Involved in the Regulation of Germline Stem Cells in Drosophila melanogaster and D. simulans
Source: G3 (Bethesda). 2015 Feb 9;5(4):583–92. doi: 10.1534/g3.114.015875 (PMC4390574; doi:10.1534/g3.114.015875)
Supplement: Supporting Information [file supp_g3.114.015875_TableS1.pdf]

Table S1 Primers used in this study

Gene	Species	Name	Sequence
cyclin A (1)	mel/sim	cycaF1	CAGTTTCCAGATCCACCAAG
cyclin A (1)	mel/sim	cycaR1	TTTAGCTTACCTCGCTCTCC
cyclin A (2)	mel/sim	cycaF2	TCTTCCAGAAGAAACATCGC
cyclin A (2)	mel/sim	cycaR2	GTATTAATATCCGGCTGCTG
nanos	mel/sim	nosF1	CAGCAACTTGGAGGGCAGTG
nanos	mel/sim	nosR1	AAACCTTCATCTGTTGCTTG
fs(1)Yb	mel	yb_sim_F1	CCTCGCTAGCCGTACATATATTAG
fs(1)Yb	mel	yb_sim_R1	GGTCAGTGGACAGTGATGAAAC
mei-P26	mel/sim	mei-p26_F2	GATGGGCTTTTGTTGAACGG
mei-P26	mel/sim	mei-p26_R2	TGCTGTTGCAGATGGTGTTG
piwi	mel/sim	piwi_F1	TTCAAAGTACTCTTTCAGTTTCC
piwi	mel/sim	piwi_R1	GTCTGGGCTAGTTTCATATATGG
pumilio (1)	mel/sim	pum_F1	CCCTACTTTCAACAGCTACAC
pumilio (1)	mel	pum_R1	CAAGCCAAGAAAGTTAACC
pumilio (1)	sim	pum_sim_R1	CAAGCCAAGAAAATTAACC
pumilio (2)	mel/sim	pum_F3	GATATTTGCTTTCCTGGAAGCC
pumilio (2)	mel/sim	pum_R3	GTCTGGGGTCTTTAGTCGG
pumilio (3)	mel	pum_F4	GGCTAAGTGGTGAATACAG
pumilio (3)	sim	pum_sim_F4	AACGTTTTAATGATAGCTTG
pumilio (3)	mel	pum_R4	GAAAATGTCACTCTGGGGAC
pumilio (3)	sim	pum_sim_R4	GAAAATGTCACTCTGGAGAC
pumilio (4)	mel	pum_F8	CATTCTCCTCTATACCTTTCC

pumilio (4)	sim	pum_sim_F8	CATTCTCTTTGATACCTCTCC
pumilio (4)	mel/sim	pum_R8	GAAGTTTCCTTTGACTGCCTG
stonewall (1)	mel/sim	stwl_F1_1	GATTGTGTGAATTGCGTTTG
stonewall (1)	mel/sim	stwl_R1_1	CTAATGGGCGATTAGTGTTAC
stonewall (2)	mel/sim	stwl_F2	CTAGCCTTATCATTTCCCTC
stonewall (2)	mel/sim	stwl_R2	CTCTTTAATCAATACTCGG
zpg	mel	zpg_F1	GTCAAACTTTACAACCGCC
zpg	sim	zpg_sim_F1	GTCAAACTTTACAAGCACC
zpg	mel	zpg_R1	GATTAAACTTGGCGTCATC
zpg	sim	zpg_sim_R1	GATTAAACTTGGTGTCATC
